# Machine learning for differentiation of lipid-poor adrenal adenoma and subclinical pheochromocytoma based on multiphase CT imaging radiomics

**DOI:** 10.1186/s12880-023-01106-2

**Published:** 2023-10-16

**Authors:** Dao-xiong Xiao, Jian-ping Zhong, Ji-dong Peng, Cun-geng Fan, Xiao-chun Wang, Xing-lin Wen, Wei-wei Liao, Jun Wang, Xiao-feng Yin

**Affiliations:** 1 Department of Medical Imaging, Ganzhou Hospital affiliated to Nanchang University, Ganzhou People’s Hospital, Ganzhou, Jiangxi province China; 2 Department of Medical Imaging, the First Affiliated Hospital of Gannan Medical University, Ganzhou, Jiangxi province China; 3 Department of Medical Imaging, Nankang District People’s Hospital, Nankang District, Ganzhou, Jiangxi province China

**Keywords:** Computed tomography, Machine learning, Radiomics, Lipid-poor adrenal adenoma, Subclinical pheochromocytoma

## Abstract

**Background:**

There is a paucity of research investigating the application of machine learning techniques for distinguishing between lipid-poor adrenal adenoma (LPA) and subclinical pheochromocytoma (sPHEO) based on radiomic features extracted from non-contrast and dynamic contrast-enhanced computed tomography (CT) scans of the abdomen.

**Methods:**

We conducted a retrospective analysis of multiphase spiral CT scans, including non-contrast, arterial, venous, and delayed phases, as well as thin- and thick-thickness images from 134 patients with surgically and pathologically confirmed. A total of 52 patients with LPA and 44 patients with sPHEO were randomly assigned to training/testing sets in a 7:3 ratio. Additionally, a validation set was comprised of 22 LPA cases and 16 sPHEO cases from two other hospitals. We used 3D Slicer and PyRadiomics to segment tumors and extract radiomic features, respectively. We then applied T-test and least absolute shrinkage and selection operator (LASSO) to select features. Six binary classifiers, including K-nearest neighbor (KNN), logistic regression (LR), decision tree (DT), random forest (RF), support vector machine (SVM), and multi-layer perceptron (MLP), were employed to differentiate LPA from sPHEO. Receiver operating characteristic (ROC) curves and area under the curve (AUC) values were compared using DeLong’s method.

**Results:**

All six classifiers showed good diagnostic performance for each phase and slice thickness, as well as for the entire CT data, with AUC values ranging from 0.706 to 1. Non-contrast CT densities of LPA were significantly lower than those of sPHEO (P < 0.001). However, using the optimal threshold for non-contrast CT density, sensitivity was only 0.743, specificity 0.744, and AUC 0.828. Delayed phase CT density yielded a sensitivity of 0.971, specificity of 0.641, and AUC of 0.814. In radiomics, AUC values for the testing set using non-contrast CT images were: KNN 0.919, LR 0.979, DT 0.835, RF 0.967, SVM 0.979, and MLP 0.981. In the validation set, AUC values were: KNN 0.891, LR 0.974, DT 0.891, RF 0.964, SVM 0.949, and MLP 0.979.

**Conclusions:**

The machine learning model based on CT radiomics can accurately differentiate LPA from sPHEO, even using non-contrast CT data alone, making contrast-enhanced CT unnecessary for diagnosing LPA and sPHEO.

## Introduction

Adrenal incidentalomas are defined as asymptomatic adrenal masses found by chance on medical imaging, and are estimated to occur in about 5–7% of adults [1]. With the advancement of imaging techniques and the popularization of abdomen CT scanning for physical examination, the detection rate of adrenal incidentalomas is increasing [2–4]. Adrenal adenomas account for the majority of adrenal incidentalomas (41–52%), followed by metastatic tumors (19%), myelolipoma (9%), and pheochromocytoma (8%) [5]. Although up to 80% of these tumors are benign, sPHEO can cause life-threatening hypertension during surgery, which can lead to serious cardiovascular events [6, 7]. However, the imaging features and non-specific clinical manifestations of sPHEO are very similar to those of LPA, which are characterized by a non-contrast CT density ≥ 10 Hounsfield unit (HU) [8, 9]. As a result, sPHEO is often misdiagnosed as LPA [10–12]. LPA accounts for about 15–30% of adrenal adenomas [13], and is not rare. Therefore, the accurate distinguishing between sPHEO and LPA prior to surgery is of great value to reduce the surgical risk of patients [14].

Currently, the recommended practice for distinguishing between sPHEO and LPA based on CT images is as follows: If the non-contrast CT density is ≥ 10 HU, a dedicated adrenal enhanced CT protocol including a 15 min delayed acquisition after contrast agent administration is recommended to evaluate absolute percentage washout (APW) and relative percentage of washout (RPW) [15]. LPA is diagnosed when APW and RPW are greater than 60% and 40%, respectively [16]. Compared with LPA, sPHEO has lower APW and RPW [10]. However, for small tumors, sPHEO could have similar washout characteristics to LPA [15]. Most importantly, due to the large number of patients examined, it is difficult for most medical units to carry out this time-consuming scan protocol with a delay of up to 15 min. In addition, a dedicated adrenal enhanced CT scan will increase the dose of X-ray radiation received by the patient and the potential risk of contrast agent allergy. Therefore, we intended to explore whether LPA and sPHEO could be distinguished based on the non-contrast CT images.

Radiomics is an advanced medical imaging analysis technique that utilizes computerized quantitative analysis to extract a wide range of image-related features, including intensity, geometry, texture, and more, from various models of medical imaging [17–19]. These features are then transformed into numerical values that can be utilized for subsequent data analysis and model building [20]. Radiomics has shown encouraging outcomes for distinguishing between different types of tumors and classifying subtypes [21]. Furthermore, radiomics can be used individually or in combination with demographic, histological, genomic, or proteomic data to address clinical problems. Highly accurate and reliable machine learning methods can be the driving force behind the successful application of radiomics in clinical practice. Radiomics has the potential to aid in the development of predictive diagnostics for personalized medicine. Studies have shown that machine learning based on non-contrast and/or contrast-enhanced CT quantitative analysis can improve the distinguishing efficiency of LPA and sPHEO [22–24].

Therefore, in this study, we developed and validated six binary models using machine learning to differentiate between LPA and sPHEO based on CT radiomic features from multiphase abdominal CT examinations. Our goal was to identify the simplest and most optimal model that can improve the preoperative diagnosis accuracy of LPA and sPHEO based on non-contrast CT images.

## Materials and methods

### Patients

We searched the institutional picture archiving and communication system (PACS) of Ganzhou People’s Hospital for medical records of patients who had surgical resection from March 2015 to November 2022, with histologically confirmed LPA and sPHEO. Patients were eligible if they had adrenal masses with a shortest diameter of at least 1 cm and an attenuation of over 10 HU on non-contrast CT. Using these criteria, we identified a total of 96 patients (52 LPA and 44 sPHEO). All 96 cases underwent CT plain scan, including both thin- and thick-slice images, with CT dynamic enhanced scan performed in 87 cases. To validate our findings, we applied the same criterion to collect data from two additional hospitals: First Affiliated Hospital of Gannan Medical University and Nankang District People’s Hospital, resulting in 38 patients (22 LPA and 16 sPHEO) for our validation dataset. All 38 cases underwent CT plain scan, with CT dynamic enhanced scan performed in 29 cases. The images in some sequences are exclusively of thick-slice.

### CT acquisition method

Patients enrolled in the study underwent similar CT examinations at all three hospitals, although different equipment and protocol were used. For example, at Ganzhou People’s Hospital, CT scans were performed using either a GE Revolution CT scanner (GE Healthcare, Milwaukee, USA) or SOMATOM Definition AS + CT scanner (Siemens, Erlangen, Germany). The non-contrast and dynamic contrast-enhanced CT examinations utilized 0.625-1.5 mm thin slice thickness and 5–6 mm thick slice thickness, 0.992:1 pitch, 120 kV, and smart mA 200–500. A total of 100 mL of non-ionic iodine contrast material was injected intravenously using a power injector at a rate of 3.0 mL/s. All patients underwent non-contrast CT and three-phase contrast-enhanced CT scans, which were obtained at 20–25 s (arterial phase), 55–60 s (venous phase), and 180 s (delayed phase) after the administration of contrast material.

### CT feature measurement

Two abdominal radiologists, Fan and Wen, with seven and six years of experience respectively, sequentially measured the shortest diameter (SD), longest diameter (LD), and attenuation on non-contrast CT (NCCT), arterial phase CT (APCT), venous phase CT (VPCT), and delayed phase CT (DPCT). The radiologists were blinded to the radiological reports and pathological findings. A consensus was reached on the measurement of conventional CT features. Each quantitative value was measured thrice, and the average was used for further analysis. The average CT value was obtained using an area of interest (ROI) with a size of one-half to two-thirds of the lesion size on thick-slice images, excluding cystic and necrotic areas of the lesion. All measurements were taken using institutional PACS or 3D Slicer.

### Radiomics acquisition and analysis

**Fig. 1:**
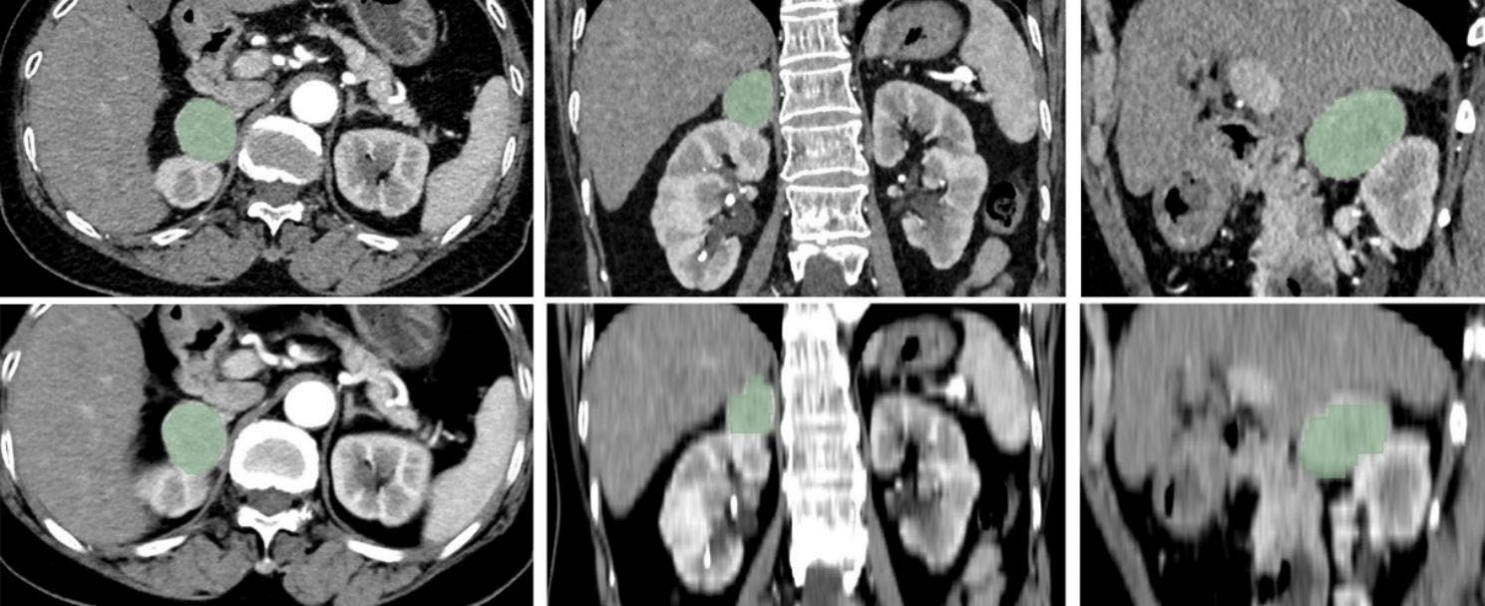
Illustration tumor segmentation of ROIs with sPHEO as an Example

To semi-automatically outline the tumor on multiple contiguous slices using 3D Slicer, first manually outline ROI along the lesion’s edge on several key slices, 1–2 mm away from it to minimize interference from surrounding fat. Then, apply the auto-fill function within the application to obtain the 3D data of the entire tumor. Tumor segmentation was performed on all sequences of each individual, including both thin- and thick-slice images. Figure 1 illustrates the segmentation of tumor ROI using sPHEO as an example. We obtained original and mask images (in NRRD format) for each series and used the PyRadiomics 3.0.1 open-source Python package (http://github.com/Radiomics/pyradiomics#readme) to extract radiomic features. Our selection criteria included first-order, morphological, and texture features, while excluding Wavelet-based features. The thin slice images were resampled to a voxel size of 1 × 1 × 1 mm, while the thick slice images were resampled to a voxel size of 5 × 5 × 5 mm when utilizing 3D slicer for tumor delineation and radiomic feature extraction. Ultimately, we assembled a dataset of 902 entries with 112 dimensions (902 rows × 112 columns). The data collected from Ganzhou People’s Hospital was split into training (498 rows × 112 columns) and test (214 rows × 112 columns) sets in a 7:3 ratio, while the data obtained from First Affiliated Hospital of Gannan Medical University and Nankang District People’s Hospital was used as the validation set (190 rows × 112 columns). All datasets were grouped based on various phases and slice thicknesses. We conducted binary classifier analysis using KNN, LR, DT, RF, SVM, and MLP for each group.

The sPHEO arterial phase thin-slice and thick-slice images are displayed in the upper and lower rows, respectively. The tumor boundaries were delineated on the transverse axial images, while the coronal and sagittal images were automatically generated by 3D Slicer.

### Statistical analysis

The Kolmogorov-Smirnov test was used to assess normality of data distribution, and the Levene test was used to evaluate homogeneity of variance. The unpaired t-test analyzed normally distributed data with equal variance, while the Mann-Whitney U test was used for non-normally distributed data. ROC curve analysis assessed performance of clinical parameters and binary classifiers in distinguishing between LPA and sPHEO. Diagnostic parameters (sensitivity, specificity, AUC) were calculated using cutoff values of clinical parameters and the classifier models with highest AUC value. ROC curves of test and validation sets were compared using DeLong’s method. Reliability was measured using intra-class correlation coefficient, with agreement classified as poor (< 0.5), moderate (0.51 to 0.8), good (0.81 to 0.89), or excellent (≥ 0.9). Significance was determined as p-value < 0.05 with all analyses conducted using Python 3.7.8, SciPy 1.7.3, scikit-learn 1.0.2, and R languages (version 4.2.2).

## Results

### Clinical characteristics

A total of 134 patients with LPA and sPHEO were included despite the absence of overt clinical manifestations. The cohort included 74 patients with LPA, ranging in age from 22 to 79 years, with a mean age of (51.54 ± 14.32) years and a male to female ratio of 29:45. Sixty patients had sPHEO, ranging in age from 18 to 78 years, with a mean age of (46.82 ± 13.19) years and a male/female ratio of 32:28. Preoperatively, 51 cases of LPA and 43 cases of sPHEO were correctly diagnosed, while 23 cases of LPA were incorrectly diagnosed as sPHEO, cyst, metastases or other disease, and 17 cases of sPHEO were incorrectly diagnosed as adrenal adenoma, hepatocellular carcinoma, mesenchymal tumor or other disease. There were no statistically significant differences in general clinical data between LPA and sPHEO, as shown in Table 1.

**Table 1:** Comparison of the general clinical profile of patients with lipid-poor adrenal adenoma and subclinical pheochromocytoma

		LPA	sPHEO	Statistics and P-values
Number of cases		74	60	
age		51.54 ± 14.32 (22–79)	46.82 ± 13.19 (18–78)	t = 1.668 p = 0.099
gender	male	29	32	X^2^ = 2.133 p = 0.144
	female	45	28	
radiologist’s diagnosis	correct	51	43	X^2^ = 0.024 p = 0.876
	incorrect	23^a^	17^b^	
	AUC	0.714		

LPA: lipid-poor adrenal adenoma; sPHEO: subclinical pheochromocytoma

a: sPHEO (15), cyst (4), metastasis (3), other disease (1)

b: adrenal adenoma (7), hepatocellular carcinoma (2), mesenchymal tumor (2), other disease (6)

### CT image characteristics

When comparing the short and long diameters of tumors, LPA was smaller than sPHEO, and the differences between them were statistically significant (p < 0.001). The mean CT values of LPA in all four phases were smaller than the CT values of sPHEO in the same phase, and the differences were all statistically significant (p < 0.05). The Jorden index was used to obtain the optimum cut-off value and corresponding sensitivity and specificity. ROC curve analysis and AUC values indicated that sPHEO was suggested when the short tumor diameter was greater than 30.78 mm, with a sensitivity of 0.743 and specificity of 0.897. Among all CT image features, the largest AUC value (0.891) was obtained for the short tumor diameter, as shown in Table 2. The Delong’s method was applied for comparative analysis of the two ROC curves. There was no statistical difference between the ROC curve of short tumor diameter and the ROC curve of other CT image features, except for the ROC curve of venous phase CT values (p > 0.05).

**Table 2:** Comparison of CT image features of lipid-poor adrenal adenoma and subclinical pheochromocytoma

CT features	LPA	sPHEO	t-/P-value	cutoff	sensitivity	specificity	AUC
Short diameter (mm)	21.26 ± 6.97	40.16 ± 14.27	t = 7.417 p < 0.001	30.78	0.743	0.897	0.891
Long diameter (mm)	27.72 ± 9.77	48.73 ± 16.84	t = 6.750 p < 0.001	39.15	0.714	0.923	0.882
NCCT (HU)	28.02 ± 7.91	36.87 ± 6.66	t = 5.461 p < 0.001	32.50	0.743	0.744	0.828
APCT (HU)	59.77 ± 19.60	79.40 ± 22.78	t = 3.951 p < 0.001	57.50	0.829	0.590	0.721
VPCT (HU)	81.64 ± 23.85	92.14 ± 18.82	t = 2.086 p = 0.041	73.00	0.857	0.385	0.614
DPCT (HU)	57.00 ± 18.90	75.06 ± 14.45	t = 4.557 p < 0.001	59.50	0.971	0.641	0.814

### Radiomic features analysis

**Fig. 2:**
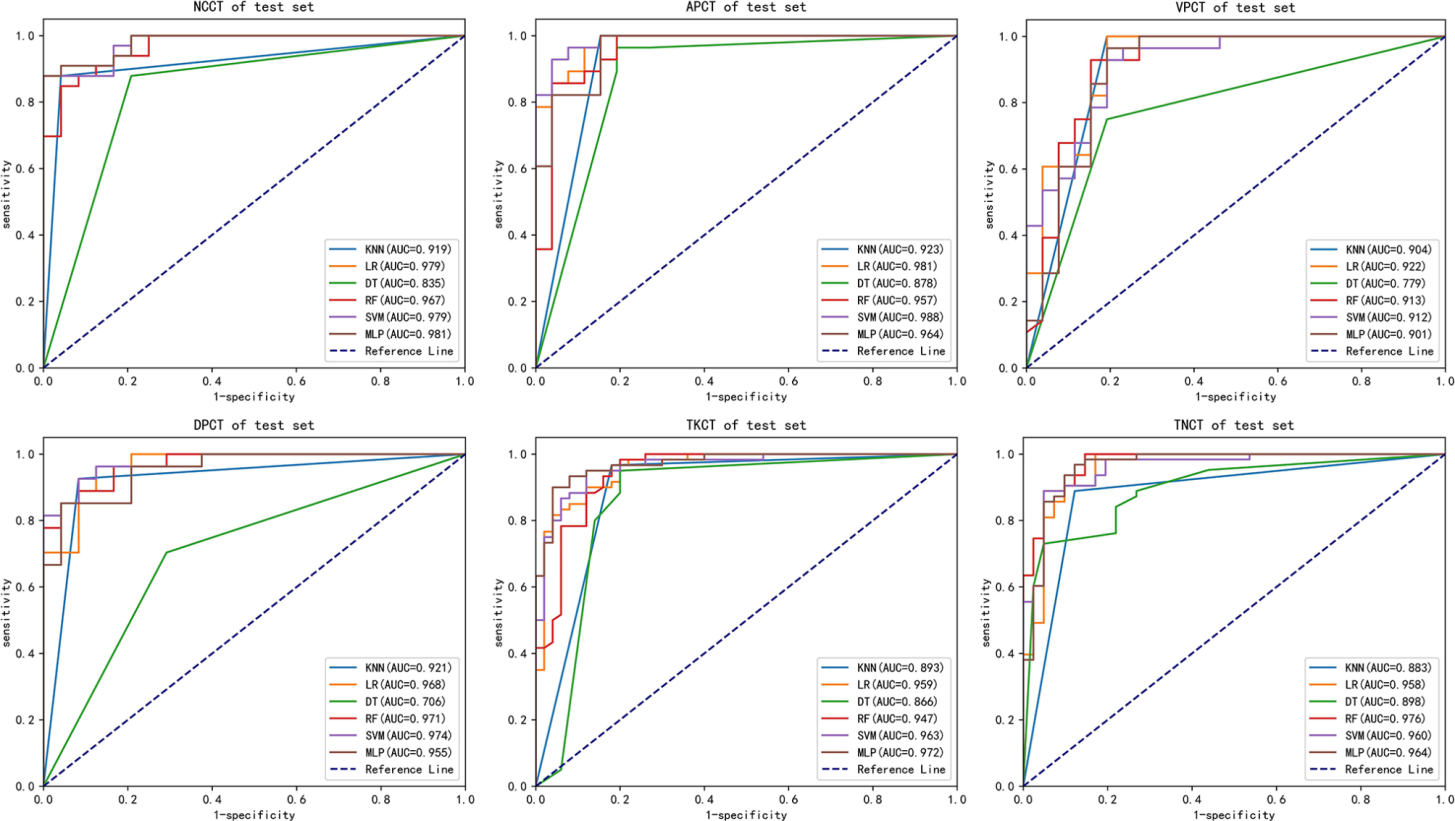
ROC curves of the six classification models in test sets with different phases and slice thicknesses

Thirty patients were randomly selected for assessing the reliability of radiomic features using intraclass correlation coefficient (ICC). Out of 112 features, 41 and 37 exhibited intra-observer and inter-observer ICC values greater than 0.8, respectively. The CT scans datasets were classified into seven groups based on unique phases and slice thicknesses: NCCT, APCT, VPCT, DPCT, thick-slice CT (TKCT), thin-slice CT (TNCT), and the multiphase and multi-slice CT (MMCT) dataset. The MMCT consists of various techniques such as non-contrast, arterial phase, venous phase, delayed phase, thick-slice, and thin-slice, which are merged into a single dataset for analysis or diagnostic purposes. Using KNN, LR, DT, RF, SVM, and MLP models, the seven groups were analyzed. ROC curves for different datasets are shown in Fig. 2, and AUC values were calculated for the test and validation datasets in Table 3. The six models showed excellent diagnostic performance with AUC values ranging from 0.706 to 1, with LR, RF, SVM, and MLP performing exceptionally well. Except for TKCT, there were no significant differences between the ROC curves of RF, SVM, and MLP on other test and validation datasets (p > 0.05). Additionally, MLP achieved high AUC values of up to 0.979 when using NCCT on the validation dataset alone, which was not significantly different from the ROC curves on other validation datasets (p > 0.05), as demonstrated in Table 4.

**Table 3:** Comparison of diagnostic efficacy and ROC curves for the six classification models on seven groups datasets

Groups of CT dataset		KNN	LR	DT	RF	SVM	MLP
NCCT	Test AUC	0.919	0.979	0.835	0.967	0.979	0.981
	Val. AUC	0.891	0.974	0.891	0.964	0.949	0.979
	P-value	0.622	0.871	0.396	0.929	0.408	0.937
APCT	Test AUC	0.923	0.981	0.878	0.957	0.988	0.964
	Val. AUC	0.978	0.96	0.953	0.97	0.973	0.96
	P-value	0.194	0.626	0.189	0.757	0.614	0.927
VPCT	Test AUC	0.904	0.922	0.779	0.913	0.912	0.901
	Val. AUC	0.933	0.968	0.909	0.969	0.972	0.98
	P-value	0.599	0.352	0.075	0.292	0.209	0.127
DPCT	Test AUC	0.921	0.968	0.706	0.971	0.974	0.955
	Val. AUC	0.887	0.975	0.875	0.98	0.975	0.952
	P-value	0.588	0.813	0.036	0.727	0.965	0.933
TKCT	Test AUC	0.893	0.959	0.866	0.947	0.963	0.972
	Val. AUC	0.979	1	0.945	1	0.999	1
	P-value	0.019	0.019	0.126	0.013	0.028	0.032
TNCT	Test AUC	0.883	0.958	0.898	0.976	0.96	0.964
	Val. AUC	0.909	0.959	0.935	0.961	0.957	0.934
	P-value	0.541	0.98	0.318	0.533	0.912	0.355
MMCT	Test AUC	0.857	0.925	0.844	0.941	0.931	0.947
	Val. AUC	0.926	0.97	0.916	0.96	0.969	0.955
	P-value	0.028	0.046	0.049	0.425	0.101	0.731

**Table 4:** Comparison of the ROC curves for NCCT in the MLP model with other validation sets

	NCCT vs. APCT	NCCT vs. VPCT	NCCT vs. DPCT	NCCT vs. MMCT
Statistics	0.445	-0.032	0.835	1.021
P-values	0.658	0.975	0.406	0.309

## Discussion

This study included 74 patients with LPA and 60 patients with sPHEO, confirmed by surgery and pathology, with no significant difference in age or gender. Conventional CT image features showed that the long and short diameters of LPA were significantly smaller than those of sPHEO (P < 0.001). The attenuation of LPA in NCCT, APCT, VPCT, and DPCT was significantly lower compared to sPHEO, consistent with previous researches [7, 25]. An optimal threshold of non-contrast CT density at 32.5HU yielded a diagnostic sensitivity of 0.743, specificity of 0.744, and AUC of 0.828. Enhanced CT (arterial, venous, and delayed phase) density yielded higher sensitivities and lower specificities compared to non-contrast CT. The highest sensitivity (0.971) was obtained by delayed phase CT density, with an optimal threshold of 59.5HU, explaining why delayed acquisition in enhanced CT examination is recommended for identifying LPA and sPHEO. However, the specificity obtained by delayed phase CT density was only 0.641, with an AUC of only 0.814, indicating that it is easy to misdiagnose sPHEO as LPA based on delayed phase CT density, potentially leading to surgical risks for patients with sPHEO. Accurately identifying LPA or sPHEO in daily imaging diagnosis remains a challenge for radiologists due to the low AUC values of these conditions, particularly when their imaging features are non-specific [7, 25]. This is true even when using both non-contrast and contrast-enhanced CT scans [14, 26].

Based on multiphase CT imaging radiomic features, we observed that the six binary models exhibited robust diagnostic performance across seven groups, with AUC values ranging from 0.706 to 1. Notably, RF, SVM, and MLP demonstrated exceptional diagnostic accuracy. Moreover, our results showed that machine learning-based on non-contrast CT features had a good effect in distinguishing LPA from sPHEO. The MLP model, using only non-contrast CT features, achieved a high AUC value. In the test set, the model demonstrated a sensitivity of 0.968 and a specificity of 0.885, while in the validation set, it achieved a sensitivity of 0.964 and a specificity of 0.962. The MLP model’s performance in diagnosing LPA and sPHEO was excellent. The performance of our study is similar to the published studies [23]. Based on radiomic features extracted from non-contrast CT images, particularly those obtained after data dimensionality reduction, a remarkable ability to differentiate between LPA and sPHEO is observed due to the robust correlation that exists between the radiomic features and conventional CT image characteristics such as shape, density, texture characteristics, etc. [13] However, compared with these studies, our study included a larger number of cases (74 LPA and 60 sPHEO) and 2 external units as the validation set, which can better verify the robustness and generalizability of the models. Yi et al. [7] also suggested that additional adrenal enhanced CT may not be necessary in the diagnosis of LPA and sPHEO.

Dynamic contrast-enhanced CT provides more comprehensive image information for the diagnosis of LPA and sPHEO compared to non-contrast CT [8, 26, 27]. Some authors suggest that radiomic features derived from contrast-enhanced CT may improve the differentiation between LPA and sPHEO [28]. However, further studies are needed to determine whether contrast-enhanced CT-based radiomic features can better distinguish between LPA and sPHEO. In general, factors affecting non-contrast CT images are relatively simple, whereas enhanced CT images are affected by many factors, including CT equipment, contrast agent type, administration method, and scan delay time for contrast enhancement. Therefore, radiomic features extracted from adrenal non-contrast CT images are more stable than those extracted from contrast-enhanced CT images. This is conducive to repeatability research and result comparison between different research institutions and the promotion and application of radiomics research results in clinical practice. Therefore, if an average AUC of 0.98 can be obtained based solely on the non-contrast CT images, as shown in the results of this paper, there is no need to obtain contrast-enhanced CT images.

However, our study also has several limitations. Firstly, the semi-automatic mapping of ROIs, particularly when delineating tumors on images with thick slice thickness, may exhibit inter-operator variability, even when performed by the same operator at different time points [29, 30]. In further research, we will consider of using a robust automatic segmentation method. Integrating the entire radiomics workflow, from image visualization to model implementation, within a single software platform would be a valuable option [31]. Secondly, differences in scanning equipment and parameters were inevitable among the three units. The use of multiple imaging scanners can result in batch effects, a well-known issue in radiomic feature analysis [32]. These effects can produce biased and unreliable outcomes, with significant consequences for patient care and clinical decision-making. Several methods have been proposed to address batch effects in radiomics, including normalization, batch correction, and harmonization [33, 34]. However, we reconstructed the slice thickness to either 1 or 5 mm to achieve a certain degree of data consistency. Finally, this study was a retrospective analysis, and we only measured surgically removed lesions. Some patients with no clinical symptoms did not receive surgery, leading to selection bias.

In conclusion, the study suggests that radiomics can effectively distinguish between LPA and sPHEO based on non-contrast CT images only. The contrast-enhanced CT may not be necessary for diagnose LPA and sPHEO.

## Data Availability

The raw datasets used during the current study are available from the corresponding author on reasonable request.

## Abbreviations

CT: Computed tomography
LPA: Lipid-poor adrenal adenoma
sPHEO: Subclinical pheochromocytoma
LASSO: Least absolute shrinkage and selection operator
KNN: k-nearest neighbor
LR: Logistic regression
DT: Decision tree
RF: Random forest
SVM: Support vector machine
MLP: Multi-layer perceptron
ROC: Receiver operating characteristic
AUC: Area under the curve
HU: Hounsfield unit
APW: Absolute percentage washout
RPW: Relative percentage of washout
PACS: Picture archiving and communication system
SD: Shortest diameter
LD: Longest diameter
NCCT: Non-contrast CT
APCT: Arterial phase CT
VPCT: Venous phase CT
DPCT: Delayed phase CT
TKCT: Thick-slice CT
TNCT: Thin-slice CT
MMCT: Multiphase and multi-slice CT
ROI: Area of interest
